# In Silico Study and Bioprospection of the Antibacterial and Antioxidant Effects of Flavone and Its Hydroxylated Derivatives

**DOI:** 10.3390/molecules22060869

**Published:** 2017-05-24

**Authors:** Camila de Albuquerque Montenegro, Gregório Fernandes Gonçalves, Abrahão Alves de Oliveira Filho, Andressa Brito Lira, Thays Thyara Mendes Cassiano, Natanael Teles Ramos de Lima, José Maria Barbosa-Filho, Margareth de Fátima Formiga Melo Diniz, Hilzeth Luna Freire Pessôa

**Affiliations:** 1 Postgraduate Program in Natural Products and Synthetic Bioactive, Federal University of Paraiba, João Pessoa 58033-455, Paraíba, Brazil; gregorio_goncalves@yahoo.com.br (G.F.G.); andressabritolira@hotmail.com (A.B.L.); jbarbosa@ltf.ufpb.br (J.M.B.-F.); margareth@reitoria.ufpb.br (M.d.F.F.M.D.); 2 Department of Pharmacy, Center Biological Sciences and Health, State University of Paraiba, Campina Grande 58429-600, Paraíba, Brazil; thaysthyaracg@hotmail.com (T.T.M.C.); teles.natanael@gmail.com (N.T.R.d.L.); 3 Academic Biological Science Unit, Health Center and Rural Technology, Federal University of Campina Grande, Patos 58708-110, Paraíba, Brazil; abrahao.farm@gmail.com; 4 Department of Pharmaceutical Sciences, Health Sciences Center, Federal University of Paraiba, João Pessoa 58059-900, Paraíba, Brazil; 5 Department of Molecular Biology, Center of Exact Sciences and Nature, Federal University of Paraíba, João Pessoa 58051-090, Paraíba, Brazil; hilzeth@gmail.com

**Keywords:** flavonoids, PASS online, antibacterials, antioxidants

## Abstract

Flavonoid compounds are widely used as natural protective species, which can act as anti-inflammatory, antioxidant, anticoagulant, antihypertensive and antitumor agents. This study set out to investigate the probable pharmacological activities, along with the antibacterial and antioxidant effects, of flavone and its hydroxy derivatives: 3-hydroxyflavone, 5-hydroxyflavone and 6-hydroxyflavone. To do so, we investigated their pharmacological characteristics, using in silico tests that indicate likelihood of activity or inactivity, with the PASS online software, and the antimicrobial potential against Gram positive and Gram negative bacteria was also analyzed, including bacteria of clinical importance. We also tested for oxidant and antioxidant potential in these molecules in the presence of reactive oxygen species (ROS) and phenylhydrazine (Ph). The results revealed the following characteristics: pharmacological activities for the flavonoids as agonists of cell membrane integrity and as permeability inhibitors, as antagonists of anaphylatoxin receptors, as inhibitors of both kinase and peroxidase, and as having both antimutagenic capacity and vaso-protective potential. All of the flavonoids exhibited moderate antibacterial activity against Gram positive and Gram negative strains, with the flavones being bactericidal at 200 μg/mL for the strains of *P. aeruginosa* ATCC 8027, *S. aureus* ATCC 25619 and *E. coli* 104; the other flavonoids revealed bacteriostatic action. The substances did not promote erythrocyte oxidation and behaved as sequestrators and antioxidants of hydrogen peroxide (H_2_O_2_) and phenylhydrazine (Ph). It was concluded that the analyzed compounds have various pharmacological activities in accordance with the predictions of PASS online, as their antibacterial and antioxidant activities were confirmed. The study also helps to consolidate the use of computational chemistry in silico tools to guide new drug search and discovery protocols.

## 1. Introduction

Flavonoids are the most common group of polyphenolic compounds and are found in a huge variety of plants [1]. They feature low molecular weight and exert effects such as modulating reactive oxygen species (ROS) levels, influencing the plant hormone auxin′s transport, and act on defense mechanisms and allelopathy [2]. They are divided into the following classes according to substitutions: flavones, flavonols, chalcones, aurones, flavanones, isoflavones, catechins and anthocyanidins. Flavones and flavonols are biosynthetically produced. It can be said that the flavonols are flavones with a hydroxyl substitute in position C3. Their analysis, synthesis, and reactions share therefore a common theoretical basis [3].

Several studies have demonstrated that flavones have anticancer activity [4,5,6,7], antioxidant [8,9,10], antitumor [11,12,13], anti-inflammatory [9,14,15,16], cardioprotective [8,17,18]; antiviral [19,20,21], neuroprotective [7,9], antibacterial [22,23,24], and induction of apoptosis in leukemic malignant cells [25]. These biological activities depend on the chemical structure and the various substituents that these molecules have; the basic backbone can undergo a number of modifications, such as glycosylation, esterification, amidation, and hydroxylation, among others that modulate the polarity, toxicity and intracellular actions of these compounds [26].

Software for in silico screening is intended to perform virtual study of molecules, and includes tools that predict theoretical selectivity and recognition for a given site of action or bioreaction, and are generally associated with large databases of small molecules for structural comparison [27,28,29]. Computational prediction models, also called predictive tools, play a prominent role in the repertoire of methodologies guiding pharmaceutical technological research. These devices are used to study existing and hypothetical substances, predicting results of pharmacological, pharmacokinetic and toxicological behavior [30,31]. Such predictive tools have already been recommended by regulatory agencies for studies of technological development in order to verify the theoretical toxicity of substances in the mammalian metabolic environment [32], in addition to allowing the creation of a database of relations between chemical structure and biological activity (SAR) [33].

**Figure 1:**
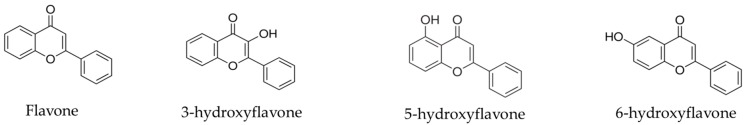
Structure of flavone and its hydroxylated derivatives.

The objective of this work was to perform in silico screening of theoretical pharmacological, pharmacokinetic, and toxicological activities as well as to investigate the antibacterial, oxidant and antioxidant effects of flavone, 3-hydroxyflavone, 5-hydroxyflavone and 6-hydroxyflavone (Figure 1); evidencing the relationship between chemical structure and observed pharmacological effect, and consolidating the use of computational chemistry to characterize substances with therapeutic potential.

## 2. Results

### 2.1. In Silico Tests—PASS Online

The online tool “PASS” provided possible biological activities for flavone, 3-hydroxyflavone, 5-hydroxyflavone, and 6-hydroxyflavone; and the results are respectively expressed in Table 1, Table 2, Table 3 and Table 4. For flavone, 37 potential biological activities were suggested; with Pa ≥ 78.7% (Pa being the probability “to be active”) (Table 1). For 3-hydroxyflavone, PASS revealed 39 likely biological activities with Pa ≥ 79.8% (Table 2). For the other hydroxylated flavonoids 5-hydroxyflavone and 6-hydroxyflavone, Table 3 and Table 4 respectively present 47 and 49 activity suggestions with respective Pa values of Pa ≥ 79.5%, and Pa ≥ 79.7%.

Among the various biological possibilities, for all the flavonoids analyzed probable agonistic action towards cell membrane integrity and inhibition of membrane permeability; a probable inhibition of kinases; a strong antimutagenic activity and metabolic influence on cytochrome P450 enzyme complexes, at times behaving as a substrate, at other times as an enzymatic inducer were indicated. In addition: inhibition of the enzymes peroxidase and oxidoreductase, and vasoprotective potential was assigned to flavone, 5-hydroxyflavone and 6-hydroxyflavone, while separately, for 5-hydroxyflavone and 6-hydroxyflavone, antitumor potential via inhibition of the Pin1 gene and increased expression of the TP53 gene was indicated.

### 2.2. Antibacterial Activity

#### 2.2.1. Determination of Minimum Inhibitory Concentration (MIC)

The flavonoids flavone, 3-hydroxyflavone, 5-hydroxyflavone and 6-hydroxyflavone have antibacterial activity against Gram positive and Gram negative strains, evidenced by the determination of the corresponding minimum inhibitory concentration (MIC_50_), which was 100 μg/mL for flavone and 200 μg/mL for the hydroxylated derivatives (Table 5 and Table 6).

#### 2.2.2. Determination of Minimum Bactericidal Concentration (MBC)

Once the antibacterial activity of flavonoids was proven, it remained to determine whether this was a result of bactericidal or bacteriostatic action. The minimum bactericidal concentration of flavone was 200 μg/mL against the strains of *P. aeruginosa* ATCC 8027, *S. aureus* ATCC 25619, and *E. coli* 104. For the other flavonoids, it was determined that against the strains tested, the antimicrobial action was bacteriostatic.

### 2.3. Oxidant and Antioxidant Activity Assay

#### 2.3.1. Evaluation of the Antioxidant Potential of these Flavonoids in Human Erythrocytes in the Presence of Reactive Oxygen Species

**Figure 2:**
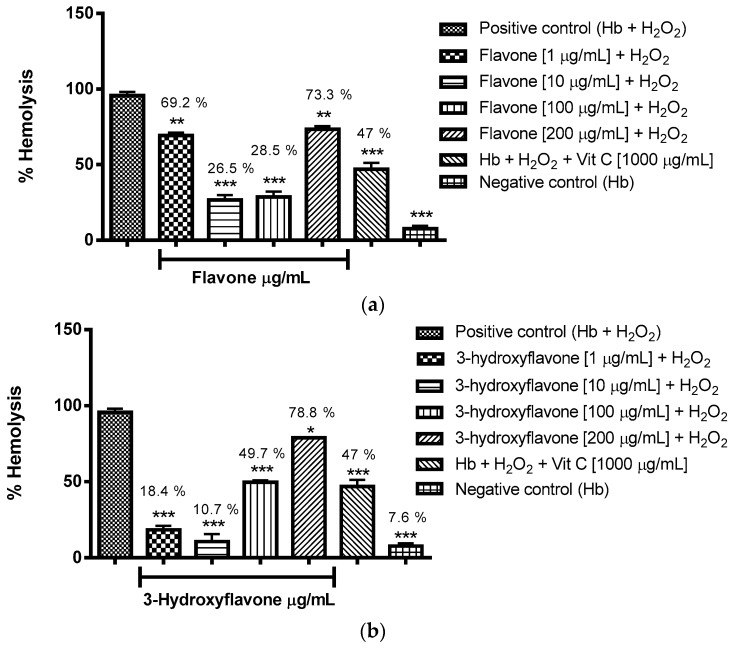

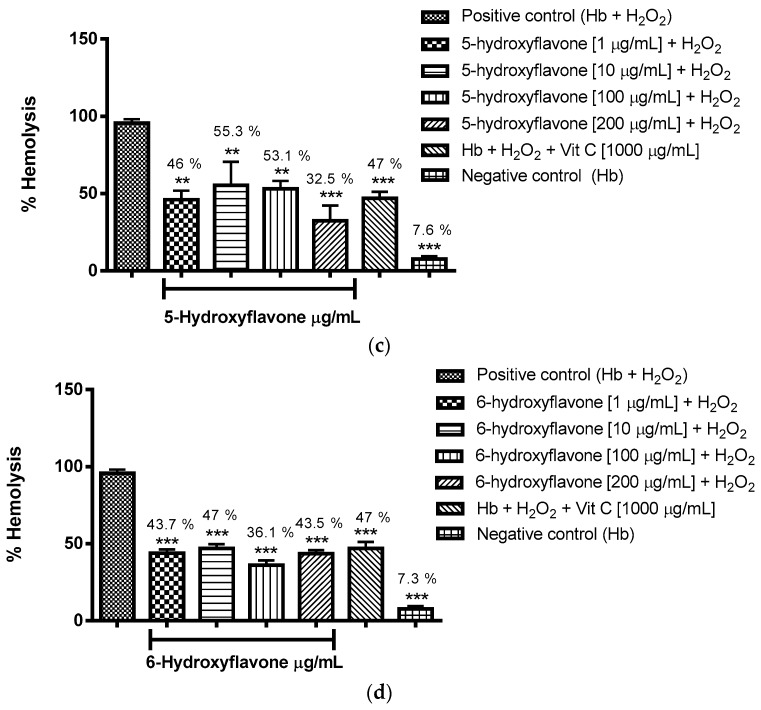
Antioxidant activity of flavonoids flavone (**a**), 3-hydroxyflavone (**b**), 5-hydroxyflavone (**c**) and 6-hydroxyflavone (**d**) against hemolysis induced by hydrogen peroxide in blood of type O+. The results are expressed as a percentage of the average in comparison to the positive control group (Hb + H_2_O_2_). Analysis by ANOVA followed by Dunnett post-test. * *p* < 0.05, ** *p* < 0.01, *** *p* < 0.001 (*n* = 3).

It was decided to evaluate antioxidant activity for concentrations of 1 to 200 μg/mL, and from the analysis of the results expressed in Figure 2a–d it was possible to assign antioxidant effect to the flavonoids flavone, 3-hydroxyflavone, 5-hydroxyflavone and 6-hydroxyflavone in all concentrations evaluated; checking reductions in hemolysis as induced by hydrogen peroxide (H_2_O_2_), as compared to the control group (Hb + H_2_O_2_).

#### 2.3.2. Assessment of the Oxidant and Antioxidant Potential of Flavonoids in Human Erythrocytes in the Presence of Phenylhydrazine

**Figure 3:**
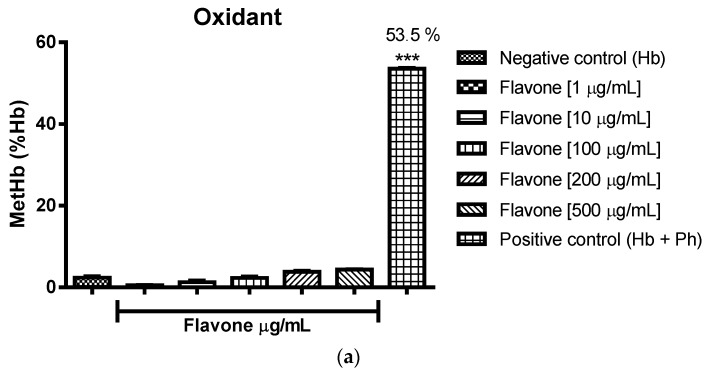

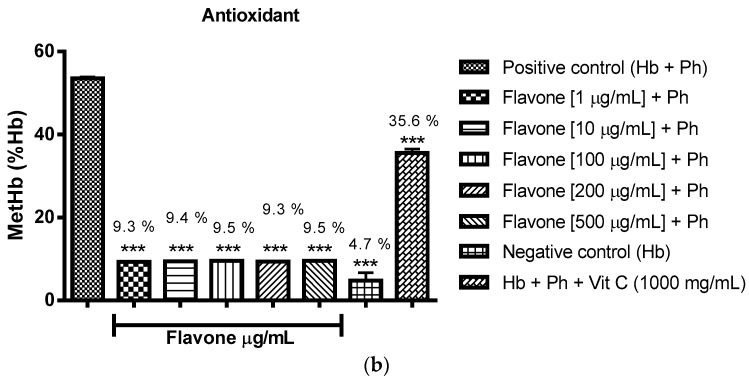
Oxidant (**a**) and antioxidant (**b**) effects of flavone on human erythrocytes. The results are expressed as a percentage of the average formation of methemoglobin (MetHb) compared to the negative control (oxidant) and positive control (antioxidant) groups. Analysis by ANOVA followed by Dunnett post-test. *** *p* < 0.001 (*n* = 3).

**Figure 4:**
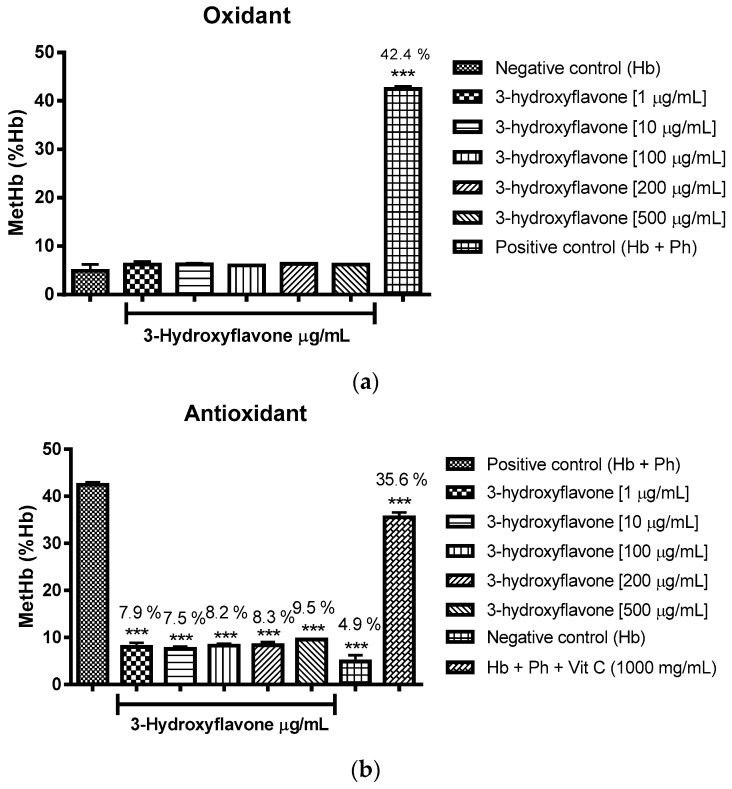
Oxidant (**a**) and antioxidant (**b**) effects of 3-hydroxyflavone on human erythrocytes. The results are expressed as a percentage of the average formation of methemoglobin (MetHb) compared to the negative control (oxidant) and positive control (antioxidant) groups. Analysis by ANOVA followed by Dunnett post-test. ** *p* < 0.001 (*n* = 3).

**Figure 5:**
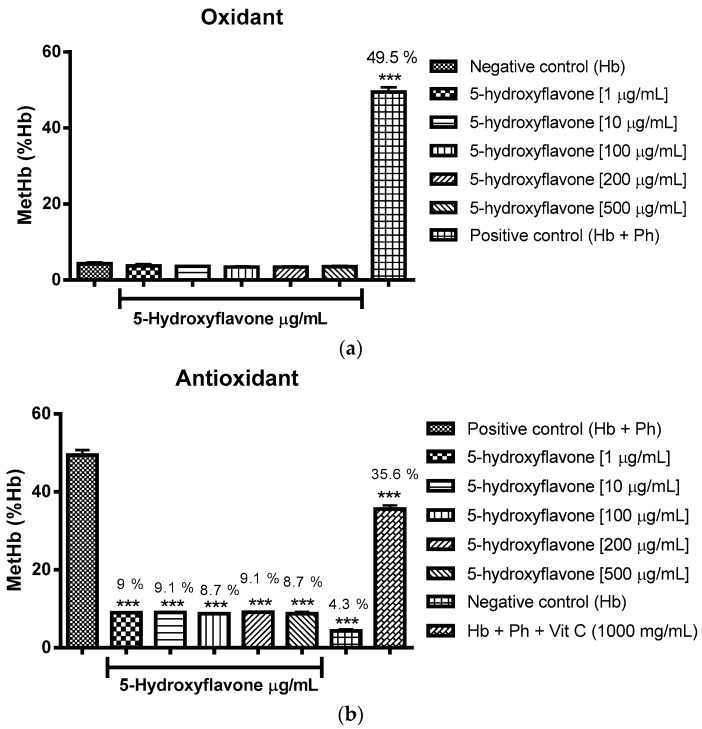
Oxidant (**a**) and antioxidant (**b**) effects of 5-hydroxyflavone on human erythrocytes. The results are expressed as a percentage of the average formation of methemoglobin (MetHb) compared to the negative control (oxidant) and positive control (antioxidant) groups. Analysis by ANOVA followed by Dunnett post-test. *** *p* < 0.001 (*n* = 3).

**Figure 6:**
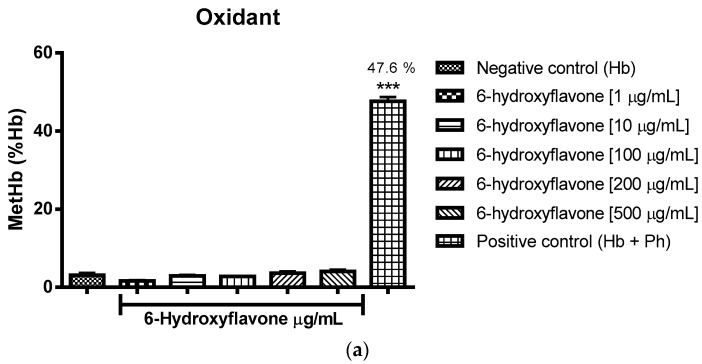

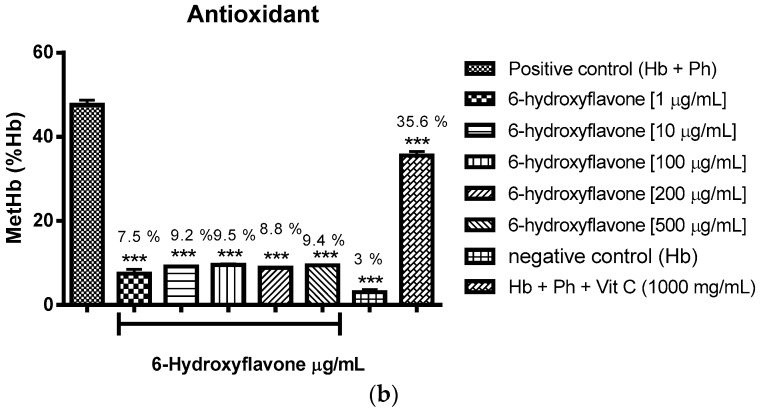
Oxidant (**a**) and antioxidant (**b**) effects of 6-hydroxyflavone on human erythrocytes. The results are expressed as a percentage of the average formation of methemoglobin (MetHb) compared to the negative control (oxidant) and positive control (antioxidant) groups. Analysis by ANOVA followed by Dunnett post-test. *** *p* < 0.001 (*n* = 3).

The oxidizing power of the flavonoids was verified through the percentage of formation of methemoglobin/hemoglobin using incubation with type O cells. It can be concluded that flavone, 3-hydroxyflavone, 5-hydroxyflavone and 6-hydroxyflavone did not induce oxidation in comparison to the negative control group (Hb-hemoglobin), as expressed in Figure 3a, Figure 4a, Figure 5a and Figure 6a.

As to the effect associated with antioxidant flavonoids, this was found through statistically significant reductions in the formation of methemoglobin/hemoglobin against phenylhydrazine as an oxidizing agent, the effect was promoted by all concentrations tested and compared to the positive control group (Hb + Ph) (Figure 3b, Figure 4b, Figure 5b and Figure 6b), performing even better than vitamin C.

It turns out that the flavonoids not only induces oxidation of hemoglobin to methemoglobin, but also protect against oxidation caused by erythrocyte phenylhydrazine.

## 3. Discussion

PASS revealed various biological possibilities: probable agonist action for cell membrane integrity and inhibition against membrane permeability; probable inhibition of kinases, antimutagenic activity and metabolic influence on cytochrome P450 enzymes, both as substrate and as inducer; in addition: flavone, 5-hydroxyflavone and 6-hydroxyflavone were assigned inhibition of peroxidase and oxidoreductase, and vasoprotective potential.

The flavonoids presented possible antitumor activity; flavone, 5-hydroxyflavone and 6-hydroxyflavone had predicted antitumor potential via inhibition of the Pin1 gene, and all three hydroxylated derivatives showed theoretical TP53 gene increases.

There are many mechanisms proposed for the effects promoted by flavonoids during the initiation and promotion stages of carcinogenicity; the principal effects are given as follows: down regulation of mutant p53 protein, cell cycle arrest, tyrosine kinase inhibition, inhibition of heat shock proteins, estrogen receptor binding capacity and inhibition of Ras proteins expression [34].

The Pin1 gene (Peptidyl-prolyl *cis*–*trans* isomerase NIMA-interacting 1) has demonstrated certain functional polymorphisms associated with cancer risk; the gene regulates conformation of phosphorylation sites, and it has been involved in multiple oncogenic signaling pathways as a critical catalyst. TP53 is a tumor suppressor gene known as “the cellular gatekeeper of growth and division”, it acts controlling cellular growth and by inducing important genes in cycle arrest and apoptosis following DNA damage [35,36].

When protein kinases (PKs) are deregulated pathological conditions may originate defects in phosphorylation leading to uncontrolled cell division, inhibition of apoptosis, and other abnormalities. Knowing this, the inhibition of these enzymes is as a promising strategy against cancer, and various studies have shown that flavonoids are capable of inhibiting a number of protein kinases from differing cellular signal pathways, such as: tyrosine kinase (PTK), serine/threonine kinases, and phosphatidylinositol 3-kinase (PI3K) [37].

One of the targets that flavones can interact with is phosphatidylinositol 3-kinase. The PI3K pathway is one of the most frequently activated signaling routes in human cancers, occurring in 30–50% of the cancerous cells [38]. The PI3K is an enzyme generally regulated by growth factors; when activated, PI3K adds a phosphate group to phosphatidylinositol 4,5-bisphosphate (PIP2), generating phosphatidylinositol 3,4,5-triphosphate (PIP3), an intra-cellular messenger. Akt, also known as protein kinase B (PKB), interacts with PIP3 and translocates to the plasma membrane, Akt is activated downstream of PI3K and has multiple targets that are extremely important in the balance control between survival and apoptosis. Some studies indicate in cancer patients, that activation of the PI3K/Akt pathway occurs because its components are targeted for amplification, mutation and translocation more frequently than any other pathway [39,40].

Lee et al. [41] demonstrated that apigenin (4′,5,7-trihydroxyflavone), a natural flavonoid, inhibits HGF-induced (Hepatocyte growth factor) which controls invasive growth of MDA-MB-231 human breast cancer cells, including their motility, scattering, migration, and invasion; through blocking the PI3K/Akt pathway. Woo et al. [42] also testing natural flavonoids investigated the effect of chrysin (5,7-dihydroxyflavone) on the apoptotic pathway in U937 human promonocytic cells; it was shown that chrysin induces apoptosis in association with the activation of caspase 3, and the Akt signal pathway plays a decisive role in chrysin-induced apoptosis in U937 cells. In the same study, they showed apoptosis increases when Akt phosphorylation in U937 cells was inhibited by the specific PI3K inhibitor; significantly LY294002, enhanced apoptosis.

The flavones tested in this work present certain structural similarities with the compounds mentioned above, and have also demonstrated a theoretical inhibitory effect on protein kinases; able to interact with PI3K/Act pathway and ERK (extracellular signal regulated kinase).

Flavone was found to have an antimicrobial effect, with an MIC of 100 μg/mL, against the strains *E. coli* 2536, *E. coli* 101, 103 *E. coli*, *E. coli* 105, *P. aeruginosa* ATCC 23243, and *S. aureus* ATCC 25619, Gram positive and Gram negative bacteria with an MIC of 200 μg/mL for the hydroxylated flavone derivatives, especially 3-hydroxyflavone that inhibited the growth of all bacterial strains tested (Table 5 and Table 6). This noted wider effect can be attributed to the presence of a hydroxyl in position 3 of the benzopyran carbon ring of the flavonoid, next to the carbonyl. It should also be noted that flavone and 5-hydroxyflavone at a concentration of 25 μg/mL showed antibacterial effect on strains of *E. coli* (103, 105, and 108). This concentration was well below that used for the standard drug chloramphenicol (100 μg/mL).

With regard to the antibacterial effect, flavone presented a bactericidal concentration of 200 μg/mL against strains of *P. aeruginosa* ATCC 8027, *S. aureus* ATCC 25619 and *E. coli* 104, while the other flavonoids were bacteriostatic at 200 μg/mL.

Ríos & Recio [43] posit avoiding evaluation tests for antibiotic activity with extract concentrations of greater than 1000 μg/mL, or 100 μg/mL for isolated substances. Antimicrobial potential is interesting when detected at concentrations below 100 μg/mL for extracts, or 10 μg/mL for active ingredients. To assign positive activity for excessively high concentrations is to be avoided. Following this evaluation criterion, the antibiotic activity of flavonoids is to be understood as moderate.

In QSAR evaluation study with flavonoids it was found that good antibacterial activity structure requirements include hydroxyl groups in positions C-3, C-5, C-7 and C-3′, the C-2, 3 unsaturated double bond and a carbonyl group at C-4 is essential, while the presence of the hydroxyl group on C-6, can lower the antibacterial activity [44]. The hydroxyl group normally works as a lead donor H, the presence of an electron donor group in conjunction with the group α, β-unsaturated, can promote electronic interactions, which decrease your bactericidal effect, observing due to the presence of variations in the structure of the compound and the replace mode [44,45]. Still, lipophilic flavonoids can also disrupt microbial membranes [34], so the high lipophilicity of flavone results in increased hydrophobic interaction by promoting a better antibacterial effect.

Lovewell et al. [46] identified for the first time that the PI3K/Akt pathway mediates the motility-driven phagocytosis of *P. aeruginosa*, and the gradual loss of bacterial flagellar motility is proportional to the degree of Akt activation in host cells, in parallel to the phagocytic susceptibility. This means that therapies using PI3K/Akt pathway inhibitors could act as bactericides, preventing phagocytosis and motility.

Since the compounds present in this work had a theoretical effect on kinases, they could act in such a way on *P. aeruginosa*. Flavonoids are able to modulate the activity of enzymes and affect the behavior of many cellular systems, exercising beneficial effects on the body [47]. Several studies have identified and attributed to flavonoids antioxidant potential. Ghasemzadeh et al. [48]. Reported that the total phenolic compounds and flavonoids in *Halia bara* possess potent antioxidant activities.

Zhao [49] suggests that the action of propolis comes from its flavonoids′ ability to interact, having lipophilic characteristics, with the bacterial plasma membrane, causing disorder. Perhaps it is through this mechanism that flavone acts in promoting bactericidal activity, since it is among the tested, the flavonoid featuring a greater cLogP (3.74), giving greater lipid solubility and allowing the same to cross the membrane. However, other mechanisms might justify the antibacterial action observed, such as inhibition of DNA gyrase, an enzyme responsible for separating the double helix during DNA replication [50,51].

To combat oxidative stress, cells and tissues have enzymatic and non-enzymatic antioxidant defense systems. However, under conditions of extreme stress such endogenous systems are not sufficient; making the support provided by antioxidants of exogenous origin necessary [31]. In this context, the red blood cells enjoy wide use in studies of oxidative stress, being a simple representative cell model. Their membranes possess the functions of other cells, such as active and passive transport and electrochemical gradients. Hydrogen peroxide (H_2_O_2_), induces changes in the form of these cells, characterized by bubbles or bulges on the cell membrane, which indicate oxidative damage [52].

From analysis of the results expressed in Figure 2a–d it was possible to assign non-concentration dependent antioxidant effect to flavone, 3-hydroxyflavone, 5-hydroxyflavone and 6-hydroxyflavone in all concentrations evaluated using reductions in hemolysis as induced by hydrogen peroxide (H_2_O_2_), this as compared to the control group (Hb + H_2_O_2_), in some situations these flavonoids showed more antioxidant potential than vitamin C.

Another protocol used to investigate the antioxidant potential of these flavonoids was the erythrocyte oxidative agent phenylhydrazine, revealing the percentage of methemoglobin formation in relation to hemoglobin (% metHb/Hb).

The Figure 3a, Figure 4a, Figure 5a and Figure 6a show respectively that flavone, 3-hydroxyflavone, 5-hydroxyflavone and 6-hydroxyflavone did not oxidize red blood cells when compared to the negative control group (Hb), and in turn presented antioxidant activity in the presence of phenylhydrazine, seeing the reduced percentages of methemoglobin formation in relation to hemoglobin (% metHb/Hb) (Figure 3b, Figure 4b, Figure 5b and Figure 6b) as compared to the positive control group (Hb + Ph).

The search for new antioxidant agents is important because the oxidative stress causes peroxidation of membrane lipids, aggression to proteins in tissues and membranes, enzymes, carbohydrates and DNA. These damages have been related to the pathogenicity of some diseases, such as atherosclerosis, neuronal degeneration, cancer, rheumatoid arthritis, diabetes mellitus, inflammation and vascular disease [53,54,55].

## 4. Materials and Methods

### 4.1. Flavonoids

The flavonoids flavone, 3-hydroxyflavone, 5-hydroxyflavone and 6-hydroxyflavone were kindly provided by Prof. Dr. José Maria Barbosa Filho of the Phytochemical Laboratory of Natural Products, of the graduate program in Natural and Synthetic Bioactive Products-CCS/UFPB, having been purchased from Sigma–Aldrich^®^ (St. Louis, MO, USA). The flavonoids were solubilized in DMSO the concentration of 10 mg/mL.

### 4.2. Bacterial Species

We evaluated Gram positive bacteria and Gram negative bacteria from the Tropical Cultures Collection (CCT, Campinas, SP, Brazil), American Type Culture Collection (ATCC, Manassas, VA) and of clinical origin, being: *Bacillus subtilis* CCT 0516, *Pseudomonas aeruginosa* ATCC 8027, *Pseudomonas aeruginosa* ATCC 23243, *Staphylococcus aureus* ATCC 25619, *Staphylococcus aureus* ATCC 25925, *Escherichia coli* 2536, *Escherichia coli* 101, *Escherichia coli* 103, *Escherichia coli* 104, *Escherichia coli* 105 and *Escherichia coli* 108.

### 4.3. Culture Medium

The bacteria were grown in Luria Bertani (LB), consisting of: yeast extract (Difco Franklin Lakes, NJ, USA) 10 g, tryptone (Difco) 5 g and NaCl (Vetec Química Fina Ltda, Duque de Caxias, RJ, Brazil), 10 g, solubilized in distilled water and autoclaved at 121 °C, 1 atm, for 15 min.

### 4.4. Preparation of the Bacterial Inoculum

Micro-organisms were inoculated in LB sterile medium and incubated at 37 °C for 24 h. The bacterial suspension was adjusted according to the standard range of 0.5 McFarland, containing 1–5 × 10^8^ CFU/mL [56,57].

### 4.5. Human Erythrocytes

The human erythrocytes of blood types A, B, O, with Rh positive and negative were obtained from bags containing concentrated of erythrocytes that would not be used for transfusion, from the Transfusional Unit of the University Hospital Lauro Wanderley—UFPB. The handling and disposal of erythrocytes followed the hospital safety standards. The experimental protocols were approved by the Research Ethics Committee/CCS/UFPB.

### 4.6. In Silico Study—PASS Online

Prediction of the spectrum of activity for substances using (PASS) online was performed to assess the overall biological potential of the organic molecule for the human organism. Based on the structures of organic compounds, the program provides simultaneous predictions of many types of biological activity. Through molecular structural analysis, the program provides a set of likely activities, giving various facets of biological action for a compound by means of interpretation of the Pa rates (probability “to be active”) and Pi (probability “to be inactive”) [31].

### 4.7. Antibacterial Activity

#### 4.7.1. Determination of Minimum Inhibitory Concentration (MIC)

Determination of MIC was performed using microdilution technique, in 96 well "U" plates as determined by Gerhardt et al. [58]. LB medium (160 μL) was distributed into all wells. Subsequently, 40 μL of solution (10 mg/mL) of each of the flavonoids was added and then half serial dilution was performed (200; 100; 50; 25; 12.5 and 6.25 μg/mL). Finally, 10 μL of each bacterial suspension (*B. subtilis* CCT 0516, *P. aeruginosa* ATCC 8027, *P. aeruginosa* ATCC 23243, *S. aureus* ATCC 25619, *S. aureus* ATCC 25925, *E. coli* 2536, *E. coli* 101, *E. coli* 103, *E. coli* 104, *E. coli* 105, *E. coli* 108) were added to the wells. The licensed antimicrobial chloramphenicol (100 μg/mL), and the vehicle DMSO were tested too. The plates were incubated at 37 °C for 24 h and bacterial growth was evidenced after addition of 20 μL of sodium resazurin solution 0.01% (*w*/*v*) (SIGMA), a colorimetric metabolic activity indicator. The MICs for the products tested were regarded as being the lowest concentration which completely inhibited bacterial growth when compared to the control group, being checked for maintaining the blue color of the resazurin. The tests were carried out in duplicate and the result was the arithmetic mean of the MIC obtained in two trials.

#### 4.7.2. Determination of Minimum Bactericidal Concentration (MBC)

Determination of MBC was performed plating de MIC dilution and at least two of the more concentrated flavonoids dilution on LB medium plus agar 1.5%. The plates were incubated at 37 °C for 24 h and bacterial growth was evaluated. The MBCs for the products tested were considered the lowest concentration in which no bacterial growth is observed.

### 4.8. Oxidant and Antioxidant Activity Assays

#### 4.8.1. Evaluation of the Antioxidant Potential of Flavonoids in Human Erythrocytes in the Presence of Reactive Oxygen Species

The experiment was carried out in accordance with a study by Bilton et al. [59], with minor modifications. Each flavonoid (1, 10, 100, 200 and 500 μg/mL) was incubated with 2 mL of a 0.5% erythrocyte suspension in 0.9% NaCl for 4 h at 25 ± 2 °C in the presence of H_2_O_2_ (40 mM). A erythrocyte suspension was used as negative control (0% cell hemolysis), and a erythrocyte suspension in the presence of H_2_O_2_ (40 mM) as positive control (100% hemolysis). Vitamin C (1000 μg/mL) was used as standard. After 4 h, the samples were centrifuged at 2500 rpm for 5 min and hemolysis was quantified by reading an aliquot of the supernatant solution using spectrophotometry at 540 nm [60]. All experiments were performed in triplicate and the results expressed as percentage of hemolysis in comparison to the positive control group (Hb + H_2_O_2_).

#### 4.8.2. Assessment of Oxidant and Antioxidant Potential for Flavonoids in Human Erythrocytes in the Presence of Phenylhydrazine

To investigate the oxidizing potential of the flavonoids a 30% erythrocytes suspension in PBS (11.35 g NaH_2_PO_4_·2H_2_O, 24.36 g Na_2_HPO_4_ and 7.18 g NaCl to 1 L, pH 7.4) supplemented with glucose (200 mg/dL), pH 7.6 was prepared. Each flavonoid (1, 10, 100, 200, 500 μg/mL) were then added to 2 mL of erythrocyte suspension and incubated for a period of 1 h under slow and steady stirring (100 rpm) at 25 ± 2 °C. Then the samples were centrifuged at 2500 rpm for 5 min and the percentage of methemoglobin (metHb) in relation to total hemoglobin (Hb) was quantified by spectrophotometry at 630 nm and 540 nm, respectively. The percentage of metHb formed was compared to values obtained in the presence the phenylhydrazine (Ph), a proven oxidizing agent [61].

To investigate the antioxidant potential, after the incubation period of 1 h, for the step described above, we added 1 mmol/L of phenylhydrazine. The solutions were aerated and kept under constant and slow agitation (100 rpm) for another 20 min to 25 ± 2 °C. After this period, the samples were centrifuged at 2500 rpm for 5 min, diluted in phosphate buffer (9 g Na_2_HPO_4_·12H_2_O; 5.7 g KH_2_PO_4_ to 1 L), and the percentage of metHb in relation to total Hb was quantified by spectrophotometry at 630 nm and 540 nm, respectively.

According to Camargo et al. [62], metHb values between 1.9% and 3.8% are considered normal while values above 4% are considered as high. The percentage of metHb formed was compared with the values obtained for vitamin C (1000 μg/mL) a proven antioxidant agent. The experiments were performed in triplicate and the results expressed as the methemoglobin formation percentage, in function of hemoglobin-metHb (% Hb), in comparison to the positive control group (Hb + Ph) [61].

## 5. Conclusions

According to the in silico approach, flavone, 3-hydroxyflavone, 5-hydroxyflavone and 6-hydroxyflavone present a wide range of pharmacological activities, especially those related to membrane integrity and physiology, inhibition of kinases and enzymes associated with oxidoreduction processes, anti-inflammatory, antimutagenic, antitumor and vasoprotective potential. The flavonoids presented good availability for oral administration and low theoretical risk of toxicity, (except flavone which presented mutagenic potential) which makes them potential candidates for future drugs.

Flavone and its hydroxylated derivatives exhibited moderate antibacterial activity, inhibiting the growth of standard and clinical Gram negative and Gram negative bacteria at different concentrations. Flavone was more effective in inhibiting the growth of Gram negative bacteria and showed bactericidal effects against the strains of *P. aeruginosa* ATCC 8027, *S. aureus* ATCC 25619 and *E. coli* 104, while the other flavonoids had bacteriostatic effect. Thus, the antibacterial effect was influenced by the electron donating group (OH) as well as variation of its position on the benzopyran ring of the flavonoids.

The flavonoids did not promote hemoglobin oxidation and had an antioxidant effect against hydrogen peroxide, suggesting a free radical sequestering action equal to phenylhydrazine, probably interfering in the formation of methemoglobin. Research like this also helps to consolidate the use of computational chemistry in silico tools to guide new drug search and discovery protocols.

## Figures and Tables

**Table 1:** Suggestions of biological activities for flavone at Pa > 78.7%—second analysis by PASS online tool.

PA	PI	Activity
0.952	0.001	Inhibitor of 4-nitrophenol 2-monooxygenase
0.952	0.003	HIF1A expression inhibitor
0.947	0.004	Agonist of membrane integrity
0.943	0.002	Inhibitor 27-hydroxycholesterol 7α-monooxygenas
0.938	0.002	Kinase inhibitor
0.933	0.002	Inhibitor of colestanetriol 26-monooxygenase
0.929	0.003	Anaphylatoxin receptor antagonist
0.914	0.003	Inhibitor of membrane permeability
0.913	0.004	Chlordecone reductase inhibitor
0.913	0.009	CYP2C12 substrate
0.903	0.002	CYP2B5 substrate
0.895	0.002	Aryl-alcohol dehydrogenase (NADP^+^) inhibitor
0.893	0.002	CYP1A2 inducer
0.874	0.014	Aspulvinone dimethylallyltransferase inhibitor
0.872	0.002	Inhibitor of P-benzoquinone reductase (NADPH)
0.857	0.004	Inhibitor of 2-dehydropantoate 2-reductase
0.857	0.006	Aldehyde oxidase inhibitor
0.854	0.003	CYP1A inducer
0.850	0.012	Methylenetetrahydrofolate reductase inhibitor (NADPH)
0.849	0.004	Vasoprotector
0.848	0.002	CYP2A4 substrate
0.829	0.002	Quercetin 2.3-dioxygenase inhibitor
0.829	0.002	Inhibitor of Leukotriene-B4 20-monooxygenase
0.820	0.005	Inhibitor of complement factor D
0.819	0.005	Alkane 1-monooxygenase inhibitor
0.814	0.003	CYP2A11 substrate
0.813	0.018	CYP2J substrate
0.813	0.022	Testosterone 17beta-dehydrogenase (NADP^+^) inhibitor
0.809	0.002	CYP1A1 inducer
0.807	0.003	MAP kinase stimulant
0.806	0.006	Inhibitor of oxidoreductase
0.804	0.005	Peroxidase inhibitor
0.803	0.003	Total ecdysone 20-monooxygenase inhibitor
0.801	0.004	Inhibitor of Pin1
0.800	0.018	mucous membrane protector
0.795	0.004	Antimutagenic
0.787	0.006	Inhibitor of nitrate reductase (cytochrome)

Pa = (probability “to be active”), Pi = (probability “to be inactive”).

**Table 2:** Suggestions of biological activities for 3-hydroxyflavone at Pa > 79.8%—second analysis by PASS online tool.

PA	PI	Activity
0.962	0.003	Agonist of membrane integrity
0.957	0.003	HIF1A expression inhibitor
0.954	0.002	Chlordecone reductase inhibitor
0.947	0.001	Aryl-alcohol dehydrogenase (NADP^+^) inhibitor
0.947	0.002	Kinase inhibitor
0.939	0.003	Inhibitor of membrane permeability
0.938	0.001	Inhibitor of *p*-benzoquinone reductase (NADPH)
0.929	0.001	Quercetin 2.3-dioxygenase inhibitor
0.922	0.002	Inhibitor of 2-dehydropantoate 2-reductase
0.917	0.002	Peroxidase inhibitor
0.896	0.009	Aspulvinone dimethylallyltransferase inhibitor
0.896	0.013	CYP2C12 substrate
0.894	0.002	MAP kinase stimulant
0.893	0.002	CYP1A inducer
0.892	0.002	Colestanetriol inhibitor 26-monooxygenase
0.889	0.002	2-Enoate reductase inhibitor
0.888	0.002	Inhibitor of 4-nitrophenol 2-monooxygenase
0.888	0.007	Inhibitor ubiquinol, cytochrome-c reductase
0.880	0.005	CYP1A substrate
0.877	0.003	Inhibitor of alcohol dehydrogenase (NADP^+^)
0.876	0.002	Inhibitor of NADPH-ferrihemoprotein reductase
0.870	0.003	Antimutagenic
0.870	0.003	Inhibitor 27-hydroxycholesterol 7α-monooxygenase
0.861	0.002	CYP1A1 inducer
0.859	0.007	Enhances expression of TP53
0.856	0.011	Methylenetetrahydrofolate reductase inhibitor (NADPH)
0.843	0.004	CYP1A1 substrate
0.843	0.008	Anaphylatoxin receptor antagonist
0.837	0.001	Inhibitor of glycerol dehydrogenase (NADP^+^)
0.827	0.003	Enhances expression of HMOX1
0.825	0.005	CYP1A2 substrate
0.819	0.004	UGT1A9 substrate
0.816	0.002	Inhibitor of 2-dehydropantolactone reductase (A-specific)
0.804	0.009	Inhibitor dehydro-L-gulonate decarboxylase
0.802	0.003	Inhibitor of β-carotene 15.15'-monooxygenase
0.800	0.005	Inhibitor of nitrate reductase (cytochrome)
0.800	0.008	Agonist of apoptosis
0.799	0.006	Alkane 1-monooxygenase inhibitor
0.798	0.021	The substrate CYP2J

Pa = (probability “to be active”), Pi = (probability “to be inactive”).

**Table 3:** Suggestions of biological activities for 5-hydroxyflavone at Pa > 79.5%—second analysis by PASS online tool.

PA	PI	Activity
0.963	0.003	Agonist of membrane integrity
0.956	0.002	Chlordecone reductase inhibitor
0.953	0.003	HIF1A expression inhibitor
0.942	0.005	Substrate of CYP2C12
0.937	0.003	Inhibitor of membrane permeability
0.935	0.002	Kinase inhibitor
0.934	0.003	Anaphylatoxin receptor antagonist
0.930	0.001	Aryl alcohol dehydrogenase inhibitor (NADP^+^)
0.923	0.004	Aldehyde oxidase inhibitor
0.921	0.002	Inhibitor of P-benzoquinone reductase (NADPH)
0.920	0.003	Inhibitor 2-dehydropantoate 2-reductase
0.909	0.002	Inhibitor of 4-nitrophenol 2-monooxygenase
0.904	0.002	Colestanetriol 26-monooxygenase inhibitor
0.903	0.001	Quercetin 2.3-dioxygenase inhibitor
0.901	0.003	Vasoprotector
0.900	0.008	Aspulvinone dimethylallyltransferase inhibitor
0.897	0.002	CYP1A inducer
0.897	0.002	Histidine kinase inhibitor
0.895	0.003	Inhibitor 27-hydroxycholesterol 7α-monooxygenase
0.888	0.003	Peroxidase inhibitor
0.887	0.008	Inhibitor of ubiquinol-cytochrome-c reductase
0.882	0.002	Antimutagenic
0.880	0.002	Inhibitor of NADPH-ferrihemoprotein reductase
0.878	0.005	CYP1A substrate
0.871	0.007	Enhances expression of TP53
0.868	0.002	CYP1A1 inducer
0.864	0.004	UGT1A6 substrate
0.854	0.003	Inhibitor of alcohol dehydrogenase (NADP^+^)
0.849	0.002	2-Enoate reductase inhibitor
0.847	0.004	Alkane 1-monooxygenase inhibitor
0.842	0.003	Enhances expression of HMOX1
0.841	0.002	Inhibitor of β-carotene 15.15′-monooxygenase
0.840	0.004	UGT1A9 substrate
0.838	0.001	Inhibitor of glycerol dehydrogenase (NADP^+^)
0.838	0.012	Anti-seborrheic
0.837	0.005	CYP1A1 substrate
0.835	0.003	CYP2B5 substrate
0.830	0.003	CYP2A4 substrate
0.827	0.003	Inhibitor of Pin1
0.818	0.002	SULT1A3 substrate
0.814	0.008	Inhibitor dihydrocholic L-gulonate decarboxylase
0.812	0.002	Inhibitor of Leukotriene-B4 20-monooxygenase
0.807	0.008	Agonist of apoptosis
0.806	0.003	Inhibitor of histamine release
0.804	0.003	Inhibitor of nitrite reductase [NAD(P)H]
0.802	0.002	Inhibitor 2-dehydropantolactone reductase (A-specific)
0.795	0.003	MAP kinase stimulant

Pa = (probability “to be active”), Pi = (probability “to be inactive”).

**Table 4:** Suggestions of biological activities for 6-hydroxyflavone at Pa > 79.2%—second analysis by PASS online tool.

PA	PI	Activity
0.957	0.003	Agonist of membrane integrity
0.949	0.002	Chlordecone reductase inhibitor
0.948	0.004	HIF1A expression inhibitor
0.945	0.004	CYP2C12 substrate
0.935	0.003	Inhibitor of membrane permeability
0.932	0.001	Aryl alcohol dehydrogenase inhibitor (NADP^+^)
0.932	0.001	Inhibitor of 4-nitrophenol 2-monooxygenase
0.929	0.003	Inhibitor aldehyde oxidase
0.924	0.002	Inhibitor of *p*-benzoquinone reductase (NADPH)
0.915	0.002	Inhibitor colestanetriol 26-monooxygenase
0.914	0.003	Kinase inhibitor
0.907	0.002	Inhibitor 27-Hydroxycolesterol 7α-monooxygenase
0.901	0.003	Inhibitor 2-2-dehydropantoate reductase
0.895	0.004	Anaphylatoxin receptor antagonist
0.894	0.002	CYP1A inducer
0.892	0.003	Peroxidase inhibitor
0.888	0.002	Antimutagenic
0.887	0.011	Aspulvinone dimethylallyltransferase inhibitor
0.884	0.002	MAP kinase stimulant
0.880	0.001	Quercetin 2.3-dioxygenase inhibitor inhibitor
0.875	0.006	Anti-seborreic
0.868	0.002	CYP1A1 inducer
0.852	0.007	Enhances expression of TP53
0.851	0.005	CYP1A substrate
0.850	0.003	Inhibitor of NADPH-ferrihemoprotein reductase
0.850	0.003	CYP2B5 substrate
0.844	0.003	Enhances expression of HMOX1
0.843	0.003	CYP2A4 substrate
0.843	0.004	UGT1A9 substrate
0.840	0.003	Inhibitor of Pin1
0.840	0.004	Vasoprotector
0.841	0.005	Expression of JAK2 inhibitor
0.833	0.003	Inhibitor of alcohol dehydrogenase (NADP^+^)
0.829	0.002	Inhibitor of leukotriene-B4 20-monooxygenase
0.828	0.005	Alkane 1-monooxygenase inhibitor
0.822	0.005	Inhibitor of oxidoreductase
0.820	0.016	Methylenetetrahydrofolate reductase inhibitor (NADPH)
0.817	0.005	Substrate of CYP1A1
0.815	0.003	Inhibitor of β-carotene 15.15′-monooxygenase
0.815	0.004	Histidine kinase inhibitor
0.809	0.002	2-Enoate reductase inhibitor
0.809	0.013	5 Hydroxytryptamine release stimulant
0.808	0.003	Inhibitor of nitrite reductase [NAD (P) H]
0.806	0.004	UGT1A6 substrate
0.805	0.017	Mucous membrane protector
0.802	0.016	Gluconate 2-dehydrogenase inhibitor
0.801	0.002	Inhibitor of Glycerol dehydrogenase (NADP^+^)
0.797	0.002	CYP1A2 inducer
0.797	0.003	Inhibitor of histamine release

Pa = (probability “to be active”), Pi = (probability “to be inactive”).

**Table 5:** Antibacterial activity (μg/mL) of flavonoids flavone, 3-hydroxyflavone, 3-hydroxyflavone and 3-hydroxyflavone against Gram-positive strains.

	Test Group	Flavone	3-Hydroxyflavone	5-Hydroxyflavone	6-Hydroxyflavone	Chloramphenicol	DMSO
Bacteria							
***B. subtilis*** **CCT 0516**		>200	200	>200	>200	100	+
***S. aureus*** **ATCC 25619**		100	100	200	200	100	+
***S. aureus*** **ATCC 25925**		200	200	>200	>200	100	+

+: Growth.

**Table 6:** Antibacterial activity (μg/mL) of flavonoids flavone, 3-hydroxyflavone, 5-hydroxyflavone and 6-hydroxyflavone against Gram-negative strains.

	Test Group	Flavone	3-Hydroxyflavone	5-Hydroxyflavone	6-Hydroxyflavone	Chloramphenicol	DMSO
Bacteria							
***P. aeruginosa*** **ATCC 8027**		200	200	>200	>200	100	+
***P. aeruginosa*** **ATCC 23243**		50	200	200	200	100	+
***E. coli*** **ATCC 2536**		100	100	200	>200	100	+
***E. coli*** **101**		100	200	200	200	100	+
***E. coli*** **103**		25	200	>200	>200	100	+
***E. coli*** **104**		200	100	200	>200	100	+
***E. coli*** **105**		25	200	200	>200	100	+
***E. coli*** **108**		200	100	25	200	100	+
